# Strategies for sample delivery for femtosecond crystallography

**DOI:** 10.1107/S2059798318017953

**Published:** 2019-02-19

**Authors:** Isabelle Martiel, Henrike M. Müller-Werkmeister, Aina E. Cohen

**Affiliations:** a Swiss Light Source, Paul Scherrer Institute, 5232 Villigen, Switzerland; b Institute of Chemistry – Physical Chemistry, University of Potsdam, Karl-Liebknecht-Strasse 24-25, 14476 Potsdam-Golm, Germany; c Stanford Synchrotron Radiation Lightsource, 2575 Sand Hill Road, Menlo Park, CA 94025, USA

**Keywords:** sample delivery, serial femtosecond crystallography, protein microcrystals, XFELs

## Abstract

Highly efficient data-collection methods are required for successful macromolecular crystallography (MX) experiments at X-ray free-electron lasers (XFELs). XFEL beamtime is scarce, and the high peak brightness of each XFEL pulse destroys the exposed crystal volume. It is therefore necessary to combine diffraction images from a large number of crystals (hundreds to hundreds of thousands) to obtain a final data set, bringing about sample-refreshment challenges that have previously been unknown to the MX synchrotron community. In view of this experimental complexity, a number of sample delivery methods have emerged, each with specific requirements, drawbacks and advantages. To provide useful selection criteria for future experiments, this review summarizes the currently available sample delivery methods, emphasising the basic principles and the specific sample requirements. Two main approaches to sample delivery are first covered: (i) injector methods with liquid or viscous media and (ii) fixed-target methods using large crystals or using microcrystals inside multi-crystal holders or chips. Additionally, hybrid methods such as acoustic droplet ejection and crystal extraction are covered, which combine the advantages of both fixed-target and injector approaches.

## Abstract 2

Strategies for sample delivery of macromolecular crystals at X-ray free-electron lasers are reviewed, covering injection methods, fixed-target approaches and hybrid methods.

## 1. Introduction   

Since their first operation in 2009, X-ray free-electron lasers (XFELs) have opened up new, previously unreachable possibilities for structural biology research in the domains of time resolution and radiation exposure. Because XFELs produce extremely bright and short (tens of femtoseconds) X-ray pulses, during a serial femtosecond crystallography (SFX) experiment the diffraction process is finished and data are recorded before the deposited X-ray dose leads to radiation damage and destruction of the sample. SFX experiments rely on this ‘diffract-before-destroy’ principle, which has proven to be a game-changer when it comes to investigating a range of scientific questions in structural biology (Neutze *et al.*, 2000 ▸). Prominent successes include the damage-free modeling of dose-sensitive metal clusters in photosystem II, allowing the determination of the oxidation states of their constituent atoms (Suga *et al.*, 2017 ▸), collective motions resulting from ligand dissociation in myoglobin (Barends *et al.*, 2015 ▸) and cytochrome *c* oxidase (Shimada *et al.*, 2017 ▸), and molecular movies of light-triggered conformation changes in bacterio­rhodopsin (Nango *et al.*, 2016 ▸) and photoactive yellow protein (Tenboer *et al.*, 2014 ▸; Pande *et al.*, 2016 ▸), as well as the study of chromophore dynamics in a switchable fluorescent protein (Coquelle *et al.*, 2017 ▸). Founding works in SFX and time-resolved SFX (TR-SFX) have paved the way for the structural biology user community to pursue future cutting-edge research.

**Figure 1:**
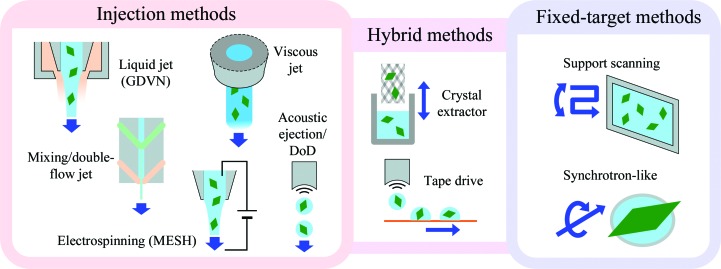
Overview of sample-delivery methods for serial femtosecond crystallography (SFX) experiments.

A key requirement for SFX is adequate delivery of the sample into the XFEL beam. The extremely bright pulses damage the sample and generate photoelectrons that quickly propagate into the surrounding material. Therefore, to most efficiently collect a data set, fresh sample must be brought into the beam between X-ray pulses, requiring a sample-exchange rate that matches the repetition rate of the XFEL source. Table 1 ▸ provides a quick reference to the beam characteristics and sample-delivery methods available currently or in the near future at all XFEL facilities worldwide. The chosen X-ray repetition rate depends on the experimental setup and the capabilities of the respective XFEL facility. For example, X-ray pulses may be delivered at rates of up to 120 Hz at the Linac Coherent Light Source (LCLS), the world’s first hard X-ray XFEL, or up to 4.5 MHz at the new European XFEL. Fast frame-rate pixel-array detectors (PADs) are used to collect diffraction images at the highest repetition rate of the facility. This paper is intended to assist XFEL users in the selection of an optimal delivery method for their project, taking into account the sample characteristics and the objectives of the experiment. Three main categories for sample delivery are detailed: crystal-injection methods, fixed-target methods and hybrid delivery methods, as outlined in Fig. 1 ▸. As ultrahigh repetition-rate (MHz) facilities come online, adaptations of the described delivery methods may be necessary to match their specific X-ray delivery patterns. Suitable modifications will be discovered as commissioning experiments commence at these facilities, and are discussed in more detail by Grünbein and Nass Kovacs (Grünbein & Nass Kovacs, 2019 ▸).

While most SFX delivery methods differ drastically from the crystal-rotation methods that are frequently used at synchrotrons (Brönnimann *et al.*, 2003 ▸), challenging microbeam experiments at the synchrotron often involve the combination of data from many microcrystals to obtain a complete data set (Rasmussen *et al.*, 2011 ▸). With the availability of brighter microbeams and faster frame-rate detectors (Casanas *et al.*, 2016 ▸; Dinapoli *et al.*, 2011 ▸) at synchrotrons, sample-delivery methods originally developed for SFX are now applied at the synchrotron for microcrystallography and dynamics studies. Serial synchrotron crystallography (SSX) is an emerging method with demonstrated success at numerous beamlines (Botha *et al.*, 2015 ▸; Martin-Garcia *et al.*, 2017 ▸; Owen *et al.*, 2017 ▸; Nogly *et al.*, 2015 ▸; Weinert *et al.*, 2017 ▸). In turn, SFX experiments have benefited from the decades-long experience of synchrotron scientists and developers. An example is fixed-target approaches that have built on existing equipment and automation (Hirata *et al.*, 2014 ▸; Cohen *et al.*, 2014 ▸). The cross-talk between XFEL and synchrotron facilities has brought about a new wave of innovation in macromolecular crystallography (MX) sample delivery, data collection and data analysis, expanding the possibilities in structural biology research.

### 1.1. Crystal-size considerations   

Choice of crystal size and sample-preparation method for SFX are primarily driven by the overall experimental goals and the sample properties. An important capability of the XFEL experiment is the ability to use very small crystals at room temperature (RT) to produce useful data sets beyond what is currently possible at the synchrotron. This makes SFX ideally suited to study samples that elude crystal growth to larger size (Mathews *et al.*, 2017 ▸; Boudes *et al.*, 2016 ▸) or to study water networks and alternative side-chain conformations within protein crystals that are disrupted by cryopreservation (Thomaston *et al.*, 2017 ▸; Keedy *et al.*, 2015 ▸). Crystallization approaches thus move from generating one perfect crystal for a traditional rotation experiment under cryoconditions to batch crystallization approaches for the preparation of large amounts of good-quality microcrystals to nanocrystals. Furthermore, homogenous reaction initiation during time-resolved studies necessitates the use of crystals that are a few micrometres in diameter or smaller, which may be more evenly exposed to pump laser pulses, light triggers or reactants during mixing experiments. While smaller crystals might show improvements in crystalline order and diffraction resolution, for the most part the observed diffraction intensity is proportional to the amount of crystalline material that is exposed. A major advantage of goniometer-based and many hybrid methods for SFX is compatibility with larger crystals and a mixture of crystal sizes. Exposing larger crystals enables the collection of stronger diffraction and higher resolution data, an approach that is well suited for detailed structural investigations of the active sites of redox-active enzymes that are highly susceptible to X-ray-induced photoreduction at the synchrotron. The use of larger crystals with goniometer setups can dramatically improve the efficiency of the SFX experiment as they may be exposed in multiple volumes and may be rotated and translated between X-ray pulses to boost the completeness.

### 1.2. Reducing background effects from the sample environment   

Independent of the sample-delivery method, background reduction is crucial for extracting the best signal to noise from microcrystal samples. The first injection experiments were performed in vacuum, for instance in the CXI hutch at LCLS (Liang *et al.*, 2015 ▸). Further, the SPB/SFX instrument at the new European XFEL has a vacuum chamber to accommodate injectors (Mancuso *et al.*, 2013 ▸). However, while a vacuum environment eliminates background air scatter, evaporative cooling may cause the carrier solution to dry or freeze, which may clog the end of the injector nozzle or produce intense background effects from ice or salt scattering. Modifications to the carrier solution, such as reducing the salt concentration or adding glycerol, are often made to mitigate these effects. Co-flow or double-flow injectors that surround the crystal-containing solution with a thin cryoprotectant-solution stream have been developed to help to address these issues (Oberthuer *et al.*, 2017 ▸; Sierra *et al.*, 2016 ▸). Moreover, dedicated chambers have been built for injection into a helium environment, such as the DAPHNIS chamber at SACLA (Tono *et al.*, 2015 ▸), the Alvra Prime station at SwissFEL (Milne *et al.*, 2017 ▸), the MICOSS chamber at PAL-XFEL (Park *et al.*, 2018 ▸) and the standard injector setup at the MFX station at LCLS (Boutet *et al.*, 2016 ▸). To reduce air scatter from the direct X-ray beam after the sample, in-atmosphere setups use post-sample flight tubes or capillaries (Roedig *et al.*, 2017 ▸; Meents *et al.*, 2017 ▸; Wiedorn *et al.*, 2017 ▸) or an X-ray beamstop, which may be translated between exposures to avoid overheating (Cohen *et al.*, 2014 ▸).

## 2. Crystal-injector methods   

The purpose of an injector is to produce a thin stream of crystals by ejecting a crystal suspension through a small orifice. The crystal stream flows orthogonally to the XFEL beam direction and they intersect at a set distance from the imaging detector. The crystal stream is interrogated by X-ray pulses at a high repetition rate and a diffraction pattern is produced each time a crystal and an X-ray pulse coincide. Crystal injectors were the first crystal-delivery method to be used for SFX (Chapman *et al.*, 2011 ▸), and are commonly used at XFEL facilities because they can efficiently deliver a large number of crystals and are well suited for time-resolved studies.

Injector techniques may be divided into low-flow and high-flow methods. While the propulsion method can strongly influence the flow rate, most low-flow injectors rely on the use of a high-viscosity carrier matrix, such as grease or LCP (lipidic cubic phase) mixtures (Weierstall *et al.*, 2014 ▸; Sugahara *et al.*, 2015 ▸). This also avoids issues of crystal settling, which can be problematic for injector methods that use low-viscosity solutions. Anti-settling devices, such as shakers or rotating arms, are often required to address these problems (Lomb *et al.*, 2012 ▸). Other useful accessories include temperature control of the sample reservoir and reservoir exchangers (Park *et al.*, 2018 ▸).

### 2.1. Sample preparation for injector studies   

To avoid clogging during injection, crystal-size filtering is often required during sample preparation or inline before injection. Uniformity of crystal size is also helpful for certain detectors, such as the CS-PAD, to avoid overloading detector pixels and to aid in data scaling (Martin-Garcia *et al.*, 2016 ▸). However, improvements in data-processing software and detector technologies are overcoming these problems. In cases where conditions for the growth of large crystals are known, microcrystal suspensions for injector studies (typically ranging between 0.5 and 10 µm in size) have been obtained by crushing larger crystals (often by centrifugation with microbeads), or by modifying the crystallization conditions to promote nucleation through seeding or increasing the protein and/or precipitant concentrations (Stevenson, Makhov *et al.*, 2014 ▸; Barnes *et al.*, 2016 ▸; Martin-Garcia *et al.*, 2016 ▸). While crystals may be combined from multiple crystallization drops, modifying conditions for larger scale batch crystallization simplifies this repetitious process. Effective protocols have been developed for batch crystallization in LCP within Hamilton gas-tight syringes, in which LCP serves as a medium for both crystallization and sample delivery (Ishchenko *et al.*, 2016 ▸). Active batch methods used for SFX involve the rapid mixing of precipitant and protein solutions (Wu *et al.*, 2015 ▸) or the dropwise addition of denser precipitant solution through the protein solution (Kupitz, Grotjohann *et al.*, 2014 ▸). As demonstrated through studies of cathepsin B, the potential of *in vivo* methods as a general crystallization method for SFX is also being explored (Redecke *et al.*, 2013 ▸; Duszenko *et al.*, 2015 ▸; Gallat *et al.*, 2014 ▸).

An exciting observation is that protein nanocrystals are ubiquitous in the granular precipitants obtained during crystallization trials (Calero *et al.*, 2014 ▸; Stevenson, Makhov *et al.*, 2014 ▸). Microcrystals that are too small (below 4 µm) for visualization using optical microscopy, including with cross-polarized light, may be detected using UV fluorescence microscopy (Desbois *et al.*, 2013 ▸), or second-order nonlinear optical imaging of chiral crystals (SONICC) may be attempted (Kissick *et al.*, 2013 ▸). For crystal optimization, the lattice order of these nanocrystals, which is predictive of diffraction quality, may be observed by TEM imaging or by powder diffraction using concentrated crystal suspensions at the synchrotron (Stevenson *et al.*, 2016 ▸; Stevenson, DePonte *et al.*, 2014 ▸; Wu *et al.*, 2015 ▸; Stevenson, Makhov *et al.*, 2014 ▸). Furthermore, many injectors may be operated at the synchrotron to test the sample and experimental setup prior to beamtime at the XFEL (Nogly *et al.*, 2015 ▸, 2016 ▸; Martin-Garcia *et al.*, 2017 ▸; Botha *et al.*, 2015 ▸; Weinert *et al.*, 2017 ▸).

### 2.2. Liquid-jet injectors   

**Figure 2:**
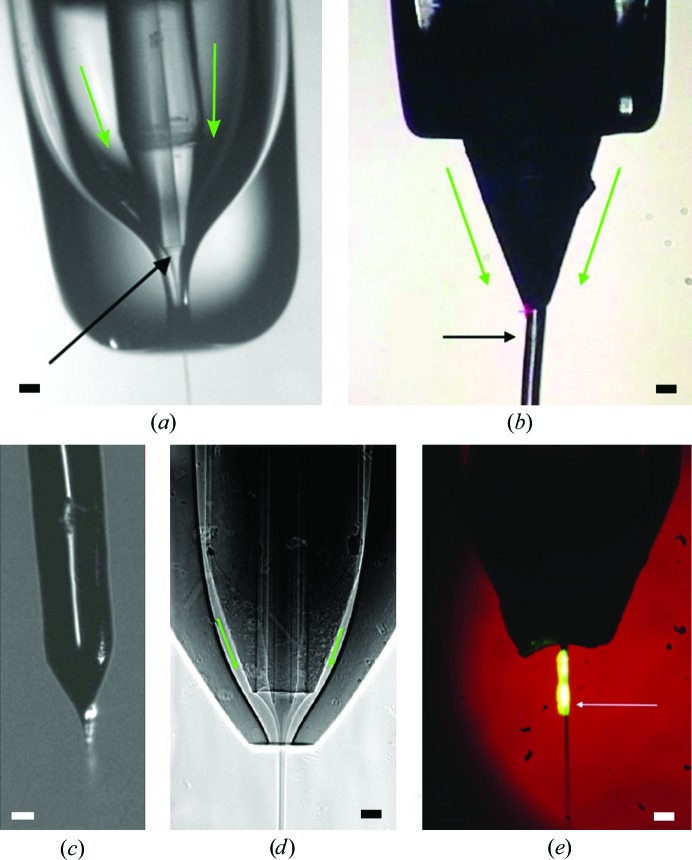
Examples of crystal-injection methods. The green arrows show the sheath helium flow. (*a*) Water jet from a GDVN, reproduced from DePonte *et al.* (2008 ▸) with the permission of IOP Publishing. The black arrow shows the flow-focusing area. (*b*) LCP injector, reproduced from Nogly *et al.* (2015 ▸). The black arrow shows the position of the XFEL beam. (*c*) Co-flow MESH injection of a photosystem II solution, with a 100 µm inner diameter of the center capillary, adapted from Sierra *et al.* (2016 ▸). Reprinted with permission from Springer Nature. Copyright (2015). (*d*) Double-flow focusing nozzle, reproduced from Oberthuer *et al.* (2017 ▸) under a Creative Commons Attribution 4.0 International Licence. (*e*) GDVN during a time-resolved experiment on PSII microcrystals, reproduced from Weierstall *et al.* (2012 ▸) with the permission of AIP Publishing. The white arrow shows the position of the XFEL beam and the green glow is the pump laser illumination. The outer glass tube of the GDVN was coned to allow the unrestricted passage of diffracted X-rays to large angles. All images are approximately on the same scale (scale bars are 50 µm).

The first crystal injector to be used at an XFEL was the gas dynamic virtual focusing nozzle (GDVN; DePonte *et al.*, 2008 ▸; Gañán-Calvo, 1998 ▸), which serves as a workhorse for SFX sample delivery (Fig. 2 ▸ *a*). The GDVN is a capillary-in-capillary device that uses a high-pressure gas sheath from the outer capillary to focus a liquid/crystal stream exiting a central capillary into a jet of a few micrometres in diameter. The GDVN is generally considered to be a rapid flow injector, as characterized by flow rates of the order of about 10–60 µl min^−1^ (Schlichting, 2015 ▸). The rapid flow rate may be well suited to probe fast reactions during mix-and-inject experiments (Calvey *et al.*, 2016 ▸) or to match the ultrafast repetition rates obtained at superconducting linac facilities such as the European XFEL and the future LCLS-II-HE, provided that jet-breakup and pressure-wave issues can be overcome (Stan *et al.*, 2016 ▸). A disadvantage of using a high-flow-rate injector with X-ray pulse rates of 120 Hz or less is high sample consumption, as large quantities of crystals pass unexposed between subsequent X-ray pulses and are thus wasted. Needle-shaped crystals can also suffer from preferred orientation along the injection-stream direction (Wojtas *et al.*, 2017 ▸), which in many cases may hinder completeness. Furthermore, as these experiments are conducted rapidly, a sample in limited supply may be consumed before any problems with the setup (alignment, detector distance *etc.*) can be detected and addressed.

Another drawback of the GDVN injector is a tendency for disruption and destabilization of the jet by crystal clogging, which often necessitates crystal-size filtering before injection. Typically, the size of compatible crystals is limited to about 5 µm in diameter. Small crystals forming clusters can also be problematic, and it is often necessary to optimize the carrier solution to avoid clumping, drying or other issues. Testing the compatibility of the crystals with the injection solution and the injection method prior to the XFEL experiment is a necessity. Furthermore, the shear forces involved during the GDVN injection process may damage delicate types of crystals, such as some membrane proteins and multi-protein complexes, as demonstrated by TEM imaging (Stevenson, DePonte *et al.*, 2014 ▸). These disadvantages are being addressed to a certain extent by subsequent developments, for instance double-flow focusing nozzles to reduce the sample consumption, stabilize the flow and reduce evaporative cooling in vacuum (Oberthuer *et al.*, 2017 ▸; Fig. 2 ▸ *d*). Advances in fabrication, particularly by two-photon polymerization 3D printing (Nelson *et al.*, 2016 ▸), have recently improved the reproducibility and stability of nozzles.

### 2.3. Viscous-media injectors   

The sample consumption during SFX experiments may be reduced by matching the crystal flow rate to the XFEL repetition rate. For this, viscous-media injectors replace the low-viscosity solutions used by GDVN-type injectors with a viscous matrix such as LCP or grease (Weierstall *et al.*, 2014 ▸; Tono *et al.*, 2015 ▸). This results in slower flow rates (0.02–0.5 µl min^−1^) that are compatible with 120 Hz pulse rates, albeit with a larger jet diameter of the order of about 50 µm (Fig. 2 ▸ *b*). Various viscous media have been used as a crystal carrier, with various characteristics and compatibilities for different types of samples. The most frequently used hydrophobic media is LCP, in which crystals, especially of membrane proteins, can be natively grown, eliminating the need to transfer these often delicate crystals into a different carrier (Nogly *et al.*, 2016 ▸). Other hydrophobic media include commercial Vaseline (Botha *et al.*, 2015 ▸) or grease (Sugahara *et al.*, 2015 ▸). Several water-based media have been proposed, which generally yield a lower scattering background and are more suitable for soluble protein crystals: agarose (Conrad *et al.*, 2015 ▸), hyaluronic acid (Sugahara *et al.*, 2016 ▸), hydroxyethylcellulose (Sugahara *et al.*, 2017 ▸), carboxymethylcellulose and Pluronic F-127 block copolymer (Kovácsová *et al.*, 2017 ▸). A popular LCP injector extrudes crystal-containing viscous media with a surrounding gas sheath for stream stabilization (Weierstall *et al.*, 2014 ▸). During experiments *in vacuo*, the composition of the matrix may need to be adjusted to avoid the negative effects of evaporative cooling (Caffrey *et al.*, 2014 ▸). The low flow rates of viscous-media injectors make them relatively straightforward to use at synchrotron facilities (Martin-Garcia *et al.*, 2017 ▸; Nogly *et al.*, 2015 ▸, 2016 ▸; Weinert *et al.*, 2017 ▸) both for data collection and sample optimization prior to XFEL beamtime.

### 2.4. Electrospinning injection   

Electrospinning injectors are a low-flow method that is compatible with both liquid and moderately viscous carrier media. A high voltage is applied to a crystal-containing solution that flows through a capillary towards the X-ray beam position. As the solution exits the capillary, the polarizable liquid surface is deformed by the electric potential, which results in the formation of a thin liquid stream that accelerates through the X-ray beam position towards a target electrode (Fig. 2 ▸ *c*). The Microfluidic Electrokinetic Sample Handling (MESH) injector has been demonstrated at an XFEL and operates with low flow rates of the order of 0.01–10 µl min^−1^ (Sierra *et al.*, 2012 ▸). Larger capillary sizes may be used compared with other injection methods (<100 µm in a vacuum, <250 µm in air), alleviating clogging issues and enabling a mixture of crystal sizes, typically between 5 and 50 µm in diameter, to be used without filtering. The MESH techniques are often considered to be a more gentle delivery method since high pressure is not applied to the sample; however, the application of an electric potential may have an influence on the observed protein structure. This injection method is compatible with a variety of low- and high-viscosity carrier media, including LCP mixtures, and in many cases the native mother liquor of the crystals is sufficient. The flow rate is governed by the carrier-fluid properties and by the applied pressure within the capillary, which may be adjusted by a syringe pump. Co-flow variants of the MESH system are available to improve performance *in vacuo* and may facilitate diffusive mixing experiments with low-viscosity solutions (Sierra *et al.*, 2016 ▸). The MESH injector is also useful at the synchrotron both for data collection and screening prior to an XFEL experiment (A. Cohen & R. Sierra, personal communication).

## 3. Fixed-target methods   

Automated positioning goniometers for crystallography are rotation- and translation-stage assemblies that precisely align crystals affixed onto a solid support (or fixed target). The ability to locate and optimally position each crystal before X-ray exposure conserves crystals and is ideally suited for scarce protein samples that require special conditions for expression and purification. Fixed-target methods also offer the possibility of *in situ* crystallization, which can avoid the sample-manipulation steps that may damage delicate crystals (Supplementary Fig. S1; Opara *et al.*, 2017 ▸; Baxter *et al.*, 2016 ▸). This is a strong advantage over injector processes, which may pose risks during crystal-size filtering steps, transfer and loading steps and by the pressures and forces involved in the injection process itself. Using fixed-target approaches, data may be collected at room temperature and controlled humidity or at cryogenic temperatures, enabling the straightforward storage and stockpiling of samples well in advance of the experiment. The crystal distribution on the holders may be examined to map crystal locations and to develop automated data-collection strategies prior to the experiment. Some holders may be robotically mounted on the goniometer, eliminating the need to enter the experimental hutch and saving time during experiments. Furthermore, the sample holders are usually compatible with the goniometer setups at microfocus synchrotron beamlines, facilitating sample screening prior to beamtime at the XFEL.

Here, we divide fixed-target methods into two groups: (i) multi-shot goniometer-based approaches using larger crystals (over 50 µm) that are exposed in multiple locations with a controlled rotation and (ii) multiple small-crystal approaches that employ supports that hold a high density of crystals and accompanying technologies for rapid positioning, where each crystal generally receives only one exposure. The development of microcrystal supports is currently a very active domain, with as yet few established commercial solutions. Here, we present concepts that are useful for microcrystal support selection, based on reported examples, rather than an exhaustive list (Supplementary Table S2). The specificities of fixed-target SFX instrumentation are also reviewed.

### 3.1. Multi-shot goniometer approaches   

Synchrotron-like goniometer experiments at an XFEL were inspired by conventional crystal-rotation methods, while integrating the requirement to constantly renew the crystal interaction volume (Hirata *et al.*, 2014 ▸; Cohen *et al.*, 2014 ▸). Larger crystals (over 50 µm) are used so that multiple volumes may be exposed. The goals of these experiments are often to avoid specific radiation damage effects to the crystal structure that may occur from even a low X-ray dose at the synchrotron (Garman, 2010 ▸) or to obtain a high-resolution structure using crystals at room temperature.

**Figure 3:**
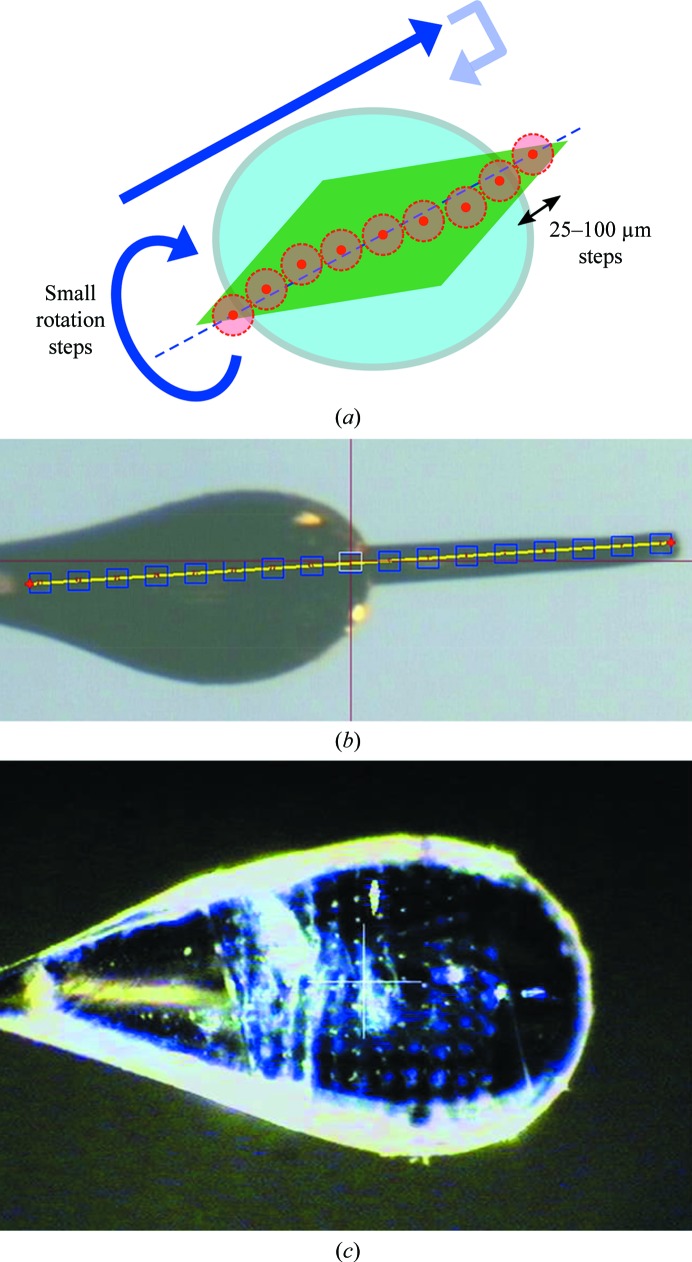
(*a*) Schematic of a synchrotron-like protein crystallography experiment at an XFEL. The crystal is translated by a step size of 25–100 µm and rotated by a fraction of its mosaicity between pulses. (*b*, *c*) Examples of samples measured with the synchrotron-like method. (*b*) A CpI [FeFe] hydrogenase crystal, with 70 µm step size, reproduced from Cohen *et al.* (2014 ▸). Copyright (2014) National Academy of Sciences. (*c*) A copper nitrite reductase crystal with 50 µm steps and 0.1° rotation steps, reproduced from Halsted *et al.* (2018 ▸).

As pictured in Figs. 3(*a*) and 3(*b*) ▸, the crystal is translated between each X-ray exposure by a step size that is just large enough to avoid the crystal volume that has undergone radiation damage from the previous exposure. Usually, the crystal is also rotated between exposures, typically by a fraction of the crystal mosaicity. The step size, which is typically between 25 and 100 µm, should be larger than the X-ray beam size and the path length of photoelectrons within the sample (Cowan & Nave, 2008 ▸). The optimal step size was selected in a semi-empirical way by Hirata *et al.* (2014 ▸), who characterized the damage propagation around the exposed volume by comparing the number of Bragg peaks present in diffraction patterns recorded with a strongly attenuated XFEL microbeam spatially scanned over the same area before and after exposure. When the full X-ray pulse is used, it will often ablate an area larger than the beam size, and hence test exposures are recommended to determine whether the consequences of this effect necessitate an increase in step size for specific crystal conditions and holders. Fig. 3 ▸(*c*) shows an example sample after exposure to the X-ray beam.

The typical size of a data set is only a few hundred images, which is considerably lower than in pure serial approaches. When using photon-flux densities that do not destroy the sample at the interaction point (by either attenuating the pulses or defocusing the beam to a larger size), additional low-intensity pulsed oscillation data can be collected from the damaged spot to help in processing. Cohen and coworkers collected 120 Hz post-exposure oscillation data at LCLS over up to 11° overlaid on a single detector frame (Cohen *et al.*, 2014 ▸). This approach mimics synchrotron rotation data collection and helps to solve indexing ambiguities when processing the first, undamaged image, as well as scaling and the estimation of reflection partiality.

Supplementary Table S1 gives an overview of synchrotron-like femtosecond experiments published in the founding years of the method. This approach has been pursued in parallel at both the SACLA and LCLS XFEL facilities. It should be noted that the data-collection rate currently exploits only a fraction of the maximum repetition rate of the respective XFELs. In these early experiments the data-collection rate was often limited by the sample-exchange time as well as the readout speed of the detector, which was chosen for its high dynamic range, as required to fully exploit the diffraction power of larger crystals. The quality and completeness of published data sets draws near to that usually observed for synchrotron data, although *de novo* phasing has not been reported to date with this method. Beyond experimental method papers (Suga *et al.*, 2014 ▸; Cohen *et al.*, 2014 ▸; Hirata *et al.*, 2014 ▸), the first user structures quickly started to appear (Zhou *et al.*, 2015 ▸; Keedy *et al.*, 2015 ▸; Chreifi *et al.*, 2016 ▸), showing that the technique is quickly coming of age.

### 3.2. Multiple microcrystal approaches   

#### 3.2.1. Sample integrity   

**Figure 4:**
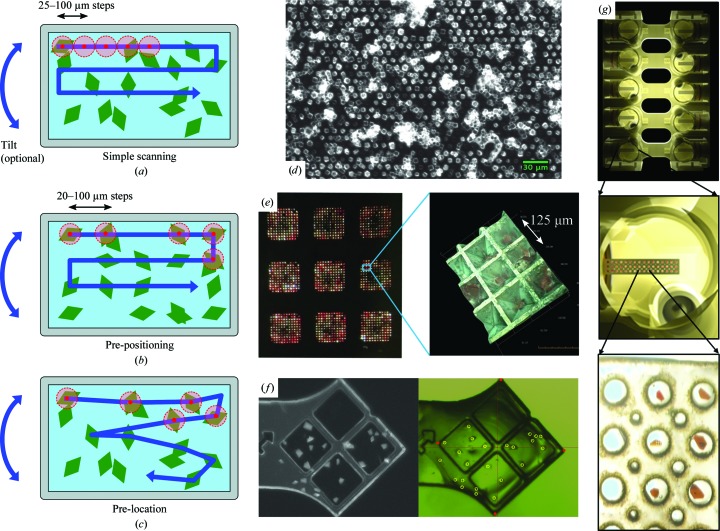
(*a*, *b*, *c*) Schematics of strategies to improve the hit rate in fixed-target serial data collection. (*a*) Linewise or raster scanning of randomly placed crystals. (*b*) Linewise scanning of pre-positioned crystals. (*c*) Targeted scanning of prelocated, randomly placed crystals. (*d*, *e*, *f*) Examples of solid supports. (*d*) Silicon chip with pre-positioned CPV18 virus crystals in holes, reproduced from Roedig *et al.* (2017 ▸) with permission from Springer Nature. Copyright (2017). (*e*) Silicon chip with myoglobin crystals pre-positioned in well shaped features, reproduced from Oghbaey *et al.* (2016 ▸). (*f*) UV microscopy image of a multi-crystal holder in the laboratory at room temperature (left) and a video-microscope view of the holder at 100 K before data collection on the MFX station at LCLS (right). The reference points are shown in red; crystal locations to be exposed are shown as yellow circles. The side dimension of the holder is 2.8 mm. (*g*) SMB *in situ* crystallization plate filled with grid sample-holder assemblies (Baxter *et al.*, 2016 ▸). The larger port holes are 0.4 mm and the length of the holder is 15.5 mm.

A major challenge in fixed-target SFX is the extreme brightness of the focused XFEL beam, which in most cases can destroy the exposed volume. When the beam is not significantly attenuated, it can create holes larger than the beam size in crystals and support materials. The physical impact, shock wave and temperature difference can also create cracks that jeopardize the overall integrity of the support. This is particularly true for brittle materials that contain internal stresses that are introduced during the fabrication process for materials such as silicon nitride or during rapid vitrification for solutions at cryogenic temperatures. Stronger interaction of all materials with the beam should also be expected at lower photon energies, resulting in more remarkable damage (Doak *et al.*, 2018 ▸). While cryocooled solutions in traditional loops may crack and fall out, shooting a sample on a thin polymer membrane, such as polycarbonate or Mylar, will reduce these effects. Empirical evidence suggests that the sample should be positioned so that the X-ray pulse first passes through the crystalline material and then through the polymer support. To minimize the risk of prematurely losing crystals, sample holders can be designed with multiple crystal ports (Baxter *et al.*, 2016 ▸; Fig. 4 ▸ *g*), window arrays (Pedrini *et al.*, 2014 ▸; Lyubimov *et al.*, 2015 ▸) or ‘compartments’ of wells (Mueller *et al.*, 2015 ▸) that isolate areas of the sample holder from the impact of previous exposures. Sealed containers at ambient temperatures pose even greater problems when liquid in the area exposed boils or outgasses, which can disrupt the position of crystals in neighboring areas. Wicking away the mother liquor surrounding the crystals is a good strategy to avoid these problems. To prevent drying out for room-temperature measurements, bare crystals may be sealed in close-fitting containers covered by a polymer foil (Oghbaey *et al.*, 2016 ▸; Mueller *et al.*, 2015 ▸; Doak *et al.*, 2018 ▸) surrounded in a viscous grease or oil such as Paratone-N (Keedy *et al.*, 2015 ▸), trapped in a sealed container (Lyubimov *et al.*, 2015 ▸) or, for experiments in atmosphere, surrounded by a controlled-humidity gas stream (Roedig *et al.*, 2017 ▸).

#### 3.2.2. Signal-to-noise optimization   

The diffraction intensity varies with the crystal volume; therefore, the small crystals used in SFX generally yield a weak diffraction signal. This is all the more true for the wide range of intrinsically weakly diffracting, challenging targets such as membrane-protein crystals. SFX solid supports and sample-preparation methods are designed to minimize background X-ray scattering to improve the overall signal-to-noise ratio. Regarding the sample support, the main parameters are the type of material and the thickness of the solid or liquid noncrystalline material in the beam path, which include the crystal-containing matrix (usually mother liquor or LCP) and the support itself.

Ultrathin supports made of silicon and silicon nitride have been investigated by several groups. Silicon nitride is an amorphous material from which membranes of submicrometre thicknesses can be fabricated by etching processes. The membranes are supported by a thicker silicon frame. The crystals can be deposited directly in their mother liquor and enclosed with a thin plastic film (Murray *et al.*, 2015 ▸) or dispersed in a viscous medium (Hunter *et al.*, 2014 ▸). However, the brittleness and high price of silicon nitride supports may limit their routine use. Microfluidic devices enabling *in situ* crystal growth within a graphene sandwich have also been reported (Sui *et al.*, 2016 ▸). Low-*Z* plastic materials represent a promising alternative owing to their versatile processing possibilities and potentially lower price. Ultrathin polymer membranes have been proposed by Feld *et al.* (2015 ▸), but their fabrication remains challenging. Most currently developed polymer-based supports, such as commercially available plastic films (Murray *et al.*, 2015 ▸; Baxter *et al.*, 2016 ▸; Doak *et al.*, 2018 ▸), employ intermediate support thicknesses of a few micrometres. This range of thickness is a satisfactory compromise between background scattering signal and ease of procurement, provided that the crystals are not too small.

Methods to remove liquid solutions surrounding the crystal have been actively pursued for background reduction (Roedig *et al.*, 2015 ▸, 2016 ▸; Mueller *et al.*, 2015 ▸) and are essentially based on the idea of the sieve. Mother-liquor removal can be elegantly combined with the trapping of crystals inside regularly spaced pores (such as pyramidal-shaped holes), which also minimize the support material along the beam path and pre-position the crystals for hit-rate optimization (Section 3.2.3). Two independent realizations of silicon-based chips with regularly spaced holes have been proposed by Roedig and coworkers (Fig. 4 ▸ *d*) and Mueller and coworkers (Fig. 4 ▸ *e*), and both are available for purchase [from Suna-Precision GmbH (http://www.suna-precision.com/Chip.html) and from the authors of the study (Owen *et al.*, 2017 ▸), respectively]. Mother liquor is removed by applying suction from below using filter paper (Roedig *et al.*, 2015 ▸) or a vacuum connection (Mueller *et al.*, 2015 ▸), while the crystals (which are larger than the holes) remain on the chip surface or are trapped inside the cavities (Mueller *et al.*, 2015 ▸; Oghbaey *et al.*, 2016 ▸). Chip loading takes place in a humid environment to prevent dehydration of the crystals. For data collection, the chips can be flash-cooled or kept at room temperature either in a humid stream (Roedig *et al.*, 2017 ▸) or between two thin plastic films (Oghbaey *et al.*, 2016 ▸). Silicon may, however, be damaged by beam tails using an unattenuated longer wavelength beam and presents a strong diffraction signal at certain angles (Doak *et al.*, 2018 ▸; Roedig *et al.*, 2015 ▸).

#### 3.2.3. Throughput and hit-rate optimization   

The use of high-density sample supports enables the collection of data from multiple crystals while minimizing the need for sample-holder exchange. The first published results at an XFEL were reported by Hunter *et al.* (2014 ▸). The support used in this proof-of-principle experiment was an array of elongated silicon nitride windows. Crystals were randomly placed within these windows, which were then scanned sequentially with an XFEL at a rate of 1 Hz (Fig. 4 ▸ *a*). The XFEL beam was sufficiently intense to damage a zone around the interaction point, which forced the researchers to impose a large step size between targets.

The throughput of the fixed-target method has been considerably improved by increasing the scanning rate and using more sophisticated crystal-location strategies. To reach optimal hit rates, the sample concentration and consumption have to be optimized as well, so that the space on the chip is efficiently used, while as little crystalline material as possible is lost in the damaged zone around the beam shots. The maximum theoretical single hit rate with a random disposition of crystals (Fig. 4 ▸ *a*) in a simple grid-like scanning scheme is about 40% (Park *et al.*, 2013 ▸) because the interaction events follow Poisson statistics. This limit of principle is shared with other random methods such as injector approaches. Progress in data processing, such as accepting multiple hits with a low number of lattices (Beyerlein, White *et al.*, 2017 ▸), as well as advances in detector technology (Mozzanica *et al.*, 2016 ▸), have contributed to the general improvement in data-collection efficiency.

To increase the hit rate in fixed-target SFX, two crystal-location strategies have been developed using high-density sample holders with controlled dimensions. The first strategy is to load crystals into well defined regularly spaced positions (Roedig *et al.*, 2015 ▸; Mueller *et al.*, 2015 ▸; Lyubimov *et al.*, 2015 ▸; Baxter *et al.*, 2016 ▸; Figs. 4 ▸ *b*, 4 ▸ *d* and 4 ▸ *e*), which are then shot sequentially. To skip the positioning and exposure of empty locations, the positions within these holders occupied by crystals may be identified in advance. This approach has been demonstrated using UV–visible microspectrometry, which is suitable for protein systems that contain a chromophore (Oghbaey *et al.*, 2016 ▸; Fig. 4 ▸ *e*). Mapping the occupied positions increases the effective hit rate tremendously (to 60–90%), but must be performed very rapidly (or offline prior to XFEL beamtime) to avoid a time overhead during the beamtime. Other more general methods to detect crystal-containing positions are second-harmonic imaging of protein crystals (SONICC) or UV tryptophan fluorescence imaging (UV imaging), which are being investigated on beamline I24 at Diamond Light Source (H. Müller-Werkmeister, personal communication).

A second strategy involves the location of randomly arranged crystals to map and translate them into position between X-ray exposures (Fig. 4 ▸ *c*). The positions of randomly oriented crystals may be identified manually using a video microscope or automatically using methods such as UV imaging (Barnes, Wu *et al.*, 2018 ▸). For example, using the standard goniometer setup on the MFX station at LCLS or on SwissMX at SwissFEL, the location of each crystal may be selected by clicking on a video view of the sample holder that is displayed within the control software interface. Once all crystals have been selected, the user clicks a start button for sequential positioning and collection. While this takes a minute or two to set up, it results in each crystal being well centered in the X-ray position. It is also possible to select a φ offset to be used when there is room for multiple exposures on crystals. Moreover, these location steps may be conducted prior to beamtime, and the relationship of the crystal positions may be saved in relation to fiducials on the sample holder. During the experiment these fiducials are clicked in a video view and the saved positions may be precisely positioned into the XFEL beam (Fig. 4 ▸ *g*). These steps may be further automated by using UV imaging methods and video analysis to automatically locate the crystals (Barnes, Wu *et al.*, 2018 ▸; Fig. 4 ▸ *f*). A UV-imaging microscope for this purpose will be added to the standard goniometer setup at the MFX station at LCLS.

### 3.3. Sample environments for fixed-target SFX   

**Figure 5:**
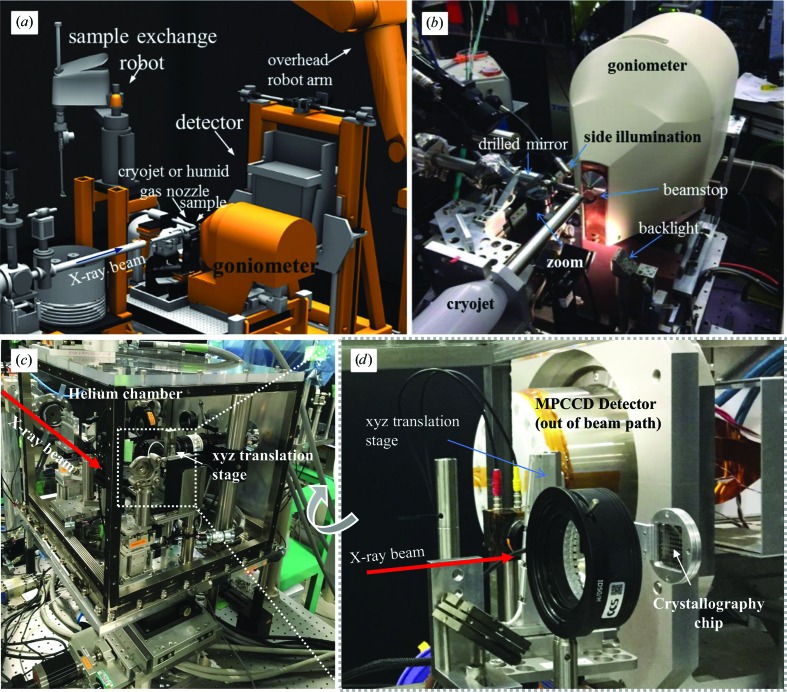
(*a*, *b*) Synchrotron-like setup using a goniometer as installed at the XPP and MFX stations at LCLS. (*a*) Drawing showing the SAM sample-exchange robot, CCD detector, cryocooler or humid gas-stream nozzle and goniometer. (*b*) Close-up photograph in data-collection mode, showing the beamstop–collimator assembly and inline viewing system (drilled mirror, zoom and illumination sources) reproduced from Cohen *et al.* (2014 ▸). Copyright (2014) National Academy of Sciences. (*c*) Overview of the scanning fixed-target setup from DLS on BL3 at SACLA within a helium chamber. (*d*) A close-up view of the setting, showing the chip and translation stage.

Equipment for multi-shot high-throughput femtosecond crystallography is typically similar to endstations developed for traditional protein crystallography at third-generation synchrotron sources, but with upgrades to more rapidly translate crystal holders and to withstand the extremely bright XFEL pulses. At the LCLS, Cohen and coworkers have installed an MX endstation (Cohen *et al.*, 2014 ▸), shown in Fig. 5 ▸, first at the XPP instrument (Chollet *et al.*, 2015 ▸) and then as the standard setup at the dedicated MFX station (Boutet *et al.*, 2016 ▸). The setup consists of a goniometer with precise rotation and positioning capacities, an inline viewing system, a cryocooler, a collimator, a beamstop and an automatic sample changer. The goniometer and automatic sample changer were originally developed for the Stanford Synchrotron Radiation Lightsource (SSRL; Russi *et al.*, 2016 ▸; Cohen *et al.*, 2002 ▸), and during the LCLS II shutdown the goniometer will be upgraded to support 120 Hz data-collection rates with randomly arranged crystals. The previous standard detector, a Rayonix MX325HE CCD, has been upgraded to a Rayonix MX340-HS-C1 for increased throughput. At SACLA, the endstation used by Suga, Hirata and coworkers (Hirata *et al.*, 2014 ▸; Suga *et al.*, 2014 ▸) is also based on former synchrotron equipment, with a single-axis goniometer, a SPACE sample changer, a cryocooler, a beamstop and a Rayonix MX225-HE CCD detector. In both cases, experiments are carried out in the atmosphere.

Addressing mapped crystals following a given path requires a setup with high positioning precision and extreme dynamic capabilities. Specialized equipment has been developed to rapidly translate microcrystal holders at the XFEL. Two proven examples of the pre-positioning approach are the Roadrunner (Roedig *et al.*, 2017 ▸) and the high-throughput fixed-target setup developed at Diamond Light Source (DLS) and used, for instance, on BL3 at SACLA (Sherrell *et al.*, 2015 ▸; Figs. 5 ▸ *c* and 5 ▸ *d*). These devices consist of an inline viewing microscope and a stack of fast and precise *x*, *y*, *z* stages that are controlled simultaneously. The motion can either be a continuous grid-like line scan as in the case of the Roadrunner, which targets future kilohertz data collection at high-repetition-rate facilities, or a fast stop-and-go motion at sequential positions as in the DLS mini-endstation. Accurate synchronization of the scanning motion to the XFEL pulse train is a key process in the data-collection workflow and has been demonstrated up to 120 Hz. At SwissFEL, the SwissMX instrument (Milne *et al.*, 2017 ▸; Pedrini *et al.*, 2016 ▸) has a custom fast scanning stage that enables continuous data collection on arbitrary trajectories at the full repetition rate of SwissFEL (100 Hz) with submicrometre accuracy to draw full advantage of crystal-prelocation strategies. At the European XFEL, *in vacuo* fixed-target scanning will be possible at the SPB/SFX instrument at a pulse-train repetition rate of 10 Hz. Large-area sample holders and an automatic sample-exchange system are foreseen.

Although XFEL setups are often still at the development level, the degree of automation tends to be high (automatic data-collection procedure, automatic sample exchange, data-processing pipelines) to make the best use of the scarce beamtime. As more user-friendly automated features are added to standard fixed-target setups for general users, the turnaround time from data collection to publication will continue to improve. For example, using the standard gonio­meter setup at the MFX station at LCLS (Boutet *et al.*, 2016 ▸), data collected during screening beamtime in run 15 resulted in a structure that was solved within a week after beamtime and was published within six months (Barnes, Gristick *et al.*, 2018 ▸), which illustrates the reduction of the technical bottleneck for end users. Furthermore, as automated methods are developed for fixed-target data collection at room temperature and controlled humidity at the XFEL, these developments will lead to a resurgence of room-temperature experiments at the synchrotron (Gati *et al.*, 2014 ▸; Owen *et al.*, 2017 ▸; Coquelle *et al.*, 2015 ▸; Meents *et al.*, 2017 ▸).

## 4. Other/hybrid approaches   

Hybrid methods have been developed to address the limitations of other sample-delivery approaches by combining the advantages of both fixed-target and injector approaches. Some of these novel delivery systems are described in the following subsections.

### 4.1. Acoustic droplet ejection and drop-on-demand   

Acoustic droplet ejection (ADE) is a liquid-manipulation method that is adapted to very small volumes, typically in the nanolitre or picolitre range, *i.e.* droplets of about 20–200 µm in diameter. It relies on the propagation of ultrasound waves emitted by a high-frequency transducer within the liquid media, resulting in the formation of a droplet train at the surface. ADE is widely used in wet biology laboratories for transferring liquids from SBS (Society for Biomolecular Screening) format plates using commercial devices such as the Labcyte Echo 550, including co-crystallization for high-throughput applications (Yin *et al.*, 2014 ▸; Teplitsky *et al.*, 2015 ▸), filling of micromeshes (Cuttitta *et al.*, 2015 ▸) and high-density grids (Baxter *et al.*, 2016 ▸). Custom devices have also been designed to deliver crystal-containing droplets onto a Kapton conveyor belt for cryocooling and data collection on the tape (Roessler *et al.*, 2013 ▸) or to directly maintain them in the synchrotron beam for fast room-temperature data collection (Tsujino & Tomizaki, 2016 ▸). At the XFEL, ADE has become a method of choice for on-demand droplet delivery in air or in a helium atmosphere. Contrary to synchrotron data collection, for which the droplets must be maintained for a short time in the beam, the XFEL can expose droplets on the fly at a fast rate. Droplet-injection rates can be matched to the repetition rate of the XFEL, thus leading to a decrease in the required sample amounts. Data collection using such a principle has been performed at LCLS using both upwards ejection and inverted geometry (Roessler *et al.*, 2016 ▸). An inkjet printing nozzle has also been used to deliver a continuous stream of droplets at SACLA (Mafuné *et al.*, 2016 ▸; Hutchison *et al.*, 2017 ▸). Conveyor-belt (also called tape-drive) setups are in use at XFELs, especially for time-resolved experiments (Fuller *et al.*, 2017 ▸), and have also become popular for serial synchrotron experiments (Beyerlein, Dierksmeyer *et al.*, 2017 ▸).

### 4.2. Crystal extractor   

**Figure 6:**
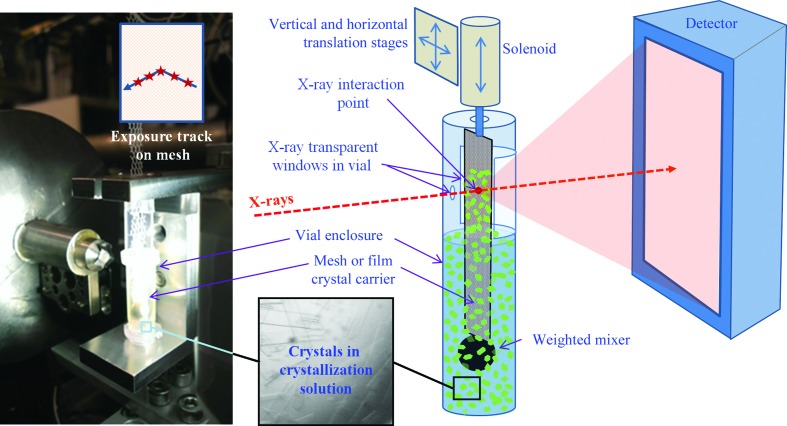
Schematic of the crystal extractor. The crystal suspension is mixed by the weighted mixer and deposited on the mesh carrier for data collection.

The crystal extractor has been developed to deliver crystals directly from mother-liquor solutions at ambient temperature and pressure, and has been demonstrated at the XFEL and the synchrotron (Mathews *et al.*, 2017 ▸). The method is compatible with a mixture of crystal sizes and is simple to set up and operate. It uses a solenoid driver to rapidly insert a crystal support, typically a mesh or thin film, into a solution of mother liquor and then quickly remove it, extracting a thin liquid film on the support that contains a random distribution of crystals (Fig. 6 ▸). The substrate is translated to position a new area of the support into the X-ray beam between exposures. At the end of a data-collection run the process is repeated, loading, extracting and exposing a fresh batch of crystals until data collection is complete or the hit rate falls off. An interesting feature of this method is that unexposed crystals are rapidly returned to the solution, with the chance of being exposed during subsequent extractions, thus dramatically reducing crystalline sample waste. A small weight at the end of the support provides positional stability and also mixes the solution, preventing the crystals from settling to the bottom. To mitigate dehydration, the setup is surrounded by a small plastic tube enclosure with an opening to allow the X-ray beam to enter and a larger opening on the opposite side covered with a thin X-ray-transparent film to let the diffracted X-rays pass through. In addition to data collection, the extractor may be useful to screen crystal mixtures for diffraction quality prior to use with a high-flow injector such as the GDVN.

### 4.3. Laser ablation   

A recent proof-of-principle experiment has demonstrated laser ablation of crystal-containing solutions as a possible approach for sample injection to match the megahertz X-ray pulse-repetition rates at the European XFEL and LCLS-II-HE (Schulz *et al.*, 2017 ▸). The approach of laser ablation is based on the ultrafast evaporation of liquid water (*i.e.* the mother liquor surrounding a protein crystal; Franjic & Miller, 2010 ▸). Water can be excited specifically with a laser beam in the mid-infrared (resonant to the H_2_O stretch vibration) and the speed of the evaporation creates sufficient force to ablate molecules and crystals suspended in the liquid into a plume which may be exposed to X-rays. In the study by Schulz *et al.* (2017 ▸), it was shown that excitation with mid-infrared laser pulses evaporates mother-liquor solutions while not disrupting the protein crystal structure. They proposed a megahertz on-demand sample-delivery setup that would synchronize ablation-based delivery of a crystal from each compartment of a crystal-containing chip to the arrival of an X-ray pulse, avoiding the speed limitations of chip-positioning hardware by directing the laser beam via reflection from a tilting mirror.

## 5. Time-resolved SFX experiments   

Proteins within crystals, including many enzymes, can retain the conformational flexibility required to perform biological processes (Rossi & Bernhard, 1970 ▸), making crystallographic studies of protein dynamics possible, as pioneered using Laue crystallography (Ihee *et al.*, 2005 ▸; Schlichting *et al.*, 1990 ▸; Schotte *et al.*, 2003 ▸). The extremely short pulse length of 5–100 fs now provided by XFELs enables dynamic crystallo­graphy on shorter time scales and using more radiation-sensitive samples, expanding these methods to investigate a wider variety of scientific questions (Neutze, 2014 ▸). Time-resolved serial femtosecond diffraction experiments (TR-SFX) use a trigger to initiate a biochemical reaction, followed by an X-ray pulse for the observation of structural changes. TR experiments are characterized by the accessible timescale, *i.e.* the delay between the trigger (or pump) and the X-ray probe. The choice of sample-delivery method will strongly depend on the trigger and the timescale of interest. Timescales from hundreds of femtoseconds to nanoseconds (or even longer) are accessible at XFELs. For adequate time resolution, the trigger must occur within a significantly shorter period than the lifetime of the interaction or intermediate of interest. Depending on the type of reaction studied, different triggering methods are required, with ultrafast pump lasers enabling the examination of the fastest reactions. Careful experimental design and prior laboratory testing is imperative to achieve homogenous reaction initiation and ensure proper timing to probe intermediate states of interest (Epp *et al.*, 2017 ▸; Sanchez-Gonzalez *et al.*, 2017 ▸). Spectroscopic monitoring is a valuable tool to monitor the progression of triggered reactions within crystals (Kern *et al.*, 2015 ▸; Cohen *et al.*, 2016 ▸). TR-SFX experiments generally demand substantial amounts of beamtime owing to the small structural changes studied and the multiple time points required for a complete study, which is sometimes called a molecular movie.

Most experiments demonstrated to date have focused on light-sensitive systems with intrinsic chromophores such as photoreceptors [bacteriorhodopsin (Nango *et al.*, 2016 ▸; Nogly *et al.*, 2018 ▸), rhodopsin and photoactive yellow protein (Pande *et al.*, 2016 ▸; Tenboer *et al.*, 2014 ▸)], light-induced ligand dynamics [heme proteins, myoglobin (Barends *et al.*, 2015 ▸) and cytochrome *c* oxidase (Shimada *et al.*, 2017 ▸)], photosynthetic reaction centers (photosystems I and II; Kupitz, Basu *et al.*, 2014 ▸; Suga *et al.*, 2017 ▸) and fluorescent proteins (Coquelle *et al.*, 2017 ▸). Light triggering covers the totality of accessible timescales, albeit with different types of pump lasers (femtosecond or nanosecond lasers). The jitter between the laser pump and X-ray probe must be well characterized for each measurement, and the fraction of chromophores excited within the proteins must be carefully adjusted based on the laser intensity, wavelength and quantum yield and the crystal size, to avoid both insufficient excitation and multi-photon overexcitation. Improvements in the coupling of pump lasers such as exposure from both sides of the sample have resulted in more complete and homogenous excitation (Shimada *et al.*, 2017 ▸). Photo­caging approaches (Lang & Chin, 2014 ▸; Tosha *et al.*, 2017 ▸) and laser-induced temperature jumps (Levantino *et al.*, 2015 ▸; Thompson *et al.*, 2018 ▸) are ways to overcome the absence of an intrinsic chromophore and to enable TR-SFX experiments on a wide range of protein systems. The majority of reported light-triggered TR-SFX experiments used liquid or viscous injection, where the time delay can be varied broadly by changing the laser frequency, the speed of the crystal jet and the physical distance between the laser and X-ray interaction regions (Fig. 2 ▸ *e*). While long time delays are easier to control, for ultrafast time delays (femtoseconds to picoseconds) the pump laser and X-ray probe have to be aligned exactly, requiring precise control of the sample position. This is where fixed-target TR-SFX has made a promising debut (Mueller *et al.*, 2015 ▸), especially in terms of the potential reduction of the required exposures, thanks to hit-rate optimization methods (Section 3.2.3). Furthermore, fixed-target time-resolved experiments have recently been demonstrated from milliseconds to seconds at a synchrotron (Schulz *et al.*, 2018 ▸). A tape-drive setup with ADE droplet deposition combined TR-SFX with X-ray emission spectroscopy (XES) to enable simultaneous diffraction and spectroscopic measurements that are useful to examine metal centers involved in enzymatic catalysis (Kern *et al.*, 2015 ▸; Fuller *et al.*, 2017 ▸). The pump and probe interaction regions can be tuned over a large distance on the tape drive to achieve the desired time delay from femtoseconds to seconds.

Hekstra *et al.* (2016 ▸) demonstrated the application of strong submicrosecond electric field pulses as a method to drive motions within single protein crystals and combined this with exposure to a train of XFEL pulses to probe the resulting conformational changes. The crystals, which were sandwiched between two glass capillaries, experienced very high fields (MV cm^−1^) that are biologically relevant, being comparable to the voltage across a cell membrane.

Serial mixing experiments are suited for the study of chemically induced protein dynamics on millisecond or longer timescales. To trigger reactions, microcrystals within a carrier solution are put in contact with a reactant solution or gas. The time resolution is limited by the diffusion of reactants into the crystals, with the uniformity of reaction initiation being strongly influenced by the crystal thickness. For fast reactions filtering may be necessary to ensure that only the smallest crystals are used. Other important factors are the concentration and the volume of the reactant solution and the crystal-containing solution, as well as the mixing geometry and flow characteristics. Rapid mixing times may be accomplished within thin concentric liquid streams such as in a rapid double-flow injector (Olmos *et al.*, 2017 ▸; Calvey *et al.*, 2016 ▸). Slower mixing times (seconds) are more easily obtained, and injectors (Stagno *et al.*, 2017 ▸) or other mixing geometries may be used such as tee-mixing of solutions subsequently deposited on tape (Beyerlein, Dierksmeyer *et al.*, 2017 ▸). Mixing timescales are generally accessible at synchrotron facilities, a prime example of developments intended for the XFEL giving rise to new synchrotron methods (Beyerlein, Dierksmeyer *et al.*, 2017 ▸).

## 6. Conclusion   

The advent of XFELs has recently brought about remarkable developments in sample-delivery methods for MX experiments. These offer exciting opportunities to link protein structure to function by enabling dynamics studies at room temperature as well as experiments with extremely radiation-sensitive crystals. While the delivery of samples in a serial manner is a requirement for XFEL experiments, the adoption of serial methods at synchrotrons is under way and will serve as a platform for testing and development in preparation for XFEL experiments. We are just beginning to realize the powerful potential of these methods, which are especially promising at synchrotron microbeam facilities when combined with fast-frame-rate PADs, such as the EIGER 16M. Developments will continue at a quick pace in view of the upcoming serial sample-delivery challenges, in particular for high-efficiency sources such as high-repetition-rate XFELs, as well as high-brilliance pink-beam beamlines at diffraction-limited fourth-generation storage rings. As technologies for structural investigations advance, studies at a range of physiological temperatures using smaller amounts of sample will be possible. However, our ability to view and understand life processes at the molecular level will continue to rely on careful experimental planning, proper sample preparation and the testing and optimization of the chosen sample-delivery and data-collection methods.

## 7. Related literature   

The following references are cited in the supporting information for this article: Casadei *et al.* (2018 ▸) and Frank *et al.* (2014 ▸).

## Supplementary Material

Supplementary Figure showing pictures of the SMB In-situ crystallization plate and Supplementary Tables summarizing relevant publications using fixed-target sample delivery.. DOI: 10.1107/S2059798318017953/ba5296sup1.pdf

## Figures and Tables

**Table 1:** XFEL sources and instruments suitable for MX experiments

XFEL source and location	Station and references	Important beam parameters†	Sample-delivery methods	Other standard equipment available
LCLS, Stanford, USA	CXI (Liang *et al.*, 2015 ▸)	0.1 and 1 µm	Jets (vacuum)	Pump–probe laser
		2.0–12.8 keV		CSPAD
		1–3 mJ		
		5–250 fs FHWM		
		120 Hz		
	MFX (Boutet *et al.*, 2016 ▸)	3 µm	Standard jet setup (He path), standard goniometer setup for fixed target (air/He)	Automatic sample changer, temperature and humidity control
		5–11 keV		
		1–3 mJ		Rayonix MX325-HS or CSPAD
		5–250 fs FWHM		
		120 Hz		
SwissFEL, Villigen, Switzerland	Alvra Prime (Milne *et al.*, 2017 ▸)	1.5 µm	Jets (vacuum/controlled atmosphere)	Pump–probe laser
		2–12.4 keV		Simultaneous spectroscopy (Von Hamos spectrometer)
		0.2–1.4 mJ		
		2–20 fs r.m.s.		Jungfrau 16M
		100 Hz		
	SwissMX at Bernina (Milne *et al.*, 2017 ▸; Pedrini *et al.*, 2016 ▸)	2–20 µm	Fixed target (air/He)	Pump–probe laser (to come)
		4.5–12.4 keV		Automatic sample changer
		0.2–1.4 mJ		Jungfrau 16M
		2–20 fs r.m.s.		
		100 Hz		
PAL-XFEL, Pohang, Republic of Korea	EH2 (experiment hutch 2) – NCI(Park *et al.*, 2018 ▸)	3–5 µm	Jets (air/He)	Pump–probe laser
		5–15 keV	Fixed target (to come)	Rayonix MX225-HS
		∼1 mJ		Jungfrau 4M (2019)
		20 fs FWHM		
		Up to 60 Hz		
SACLA, Hyogo, Japan	BL3 and BL2 (Tono *et al.*, 2013 ▸)	1–5 µm	Jets (air/He), fixed target (air)	Pump–probe laser
		4–20 keV		Automatic sample changer
		0.5 mJ at 10 keV		Rayonix MX225-HS MPCCD
		2–10 fs FWHM		
		30 Hz, up to 60 Hz		
European XFEL, Hamburg, Germany	SPB/SFX (Altarelli & Mancuso, 2014 ▸; Tschentscher *et al.*, 2017 ▸; Bean *et al.*, 2016 ▸)	1 µm or ∼0.1 µm upstream, 1 µm downstream	Liquid/aerosol/gas injection systems (vacuum), fixed-target scanner (He)	Pump–probe laser
				AGIPD 1M and 4M, Jungfrau 4M (2019)
		0.5 mJ at 9 keV		
		5–300 fs		
		10 Hz train rate, 4.5 MHz in train, 2700 pulses per train		

† Focus size, photon energy, pulse intensity, pulse duration and maximum repetition rate. All of the reported beam parameters are indicative and may not be obtained simultaneously in a given beamline configuration. The beam parameters listed for the European XFEL, SwissFEL and PAL-XFEL facilities are the design parameters, which may not yet have been achieved. Not all detectors can collect at the full repetition rate of the facility.
