# Supplementary material for: Strategies for sample delivery for femtosecond crystallography
Source: Acta Crystallogr D Struct Biol. 2019 Feb 19;75(Pt 2):160–77. doi: 10.1107/S2059798318017953 (PMC6400256; doi:10.1107/S2059798318017953)
Supplement: Supplementary file 1 [file d-75-00160-sup1.pdf]

STRUCTURAL
BIOLOGY

Volume 75 (2019)

Supporting information for article:

Strategies for sample delivery for femtosecond crystallography

Isabelle Martiel, Henrike M. Müller-Werkmeister and Aina E. Cohen

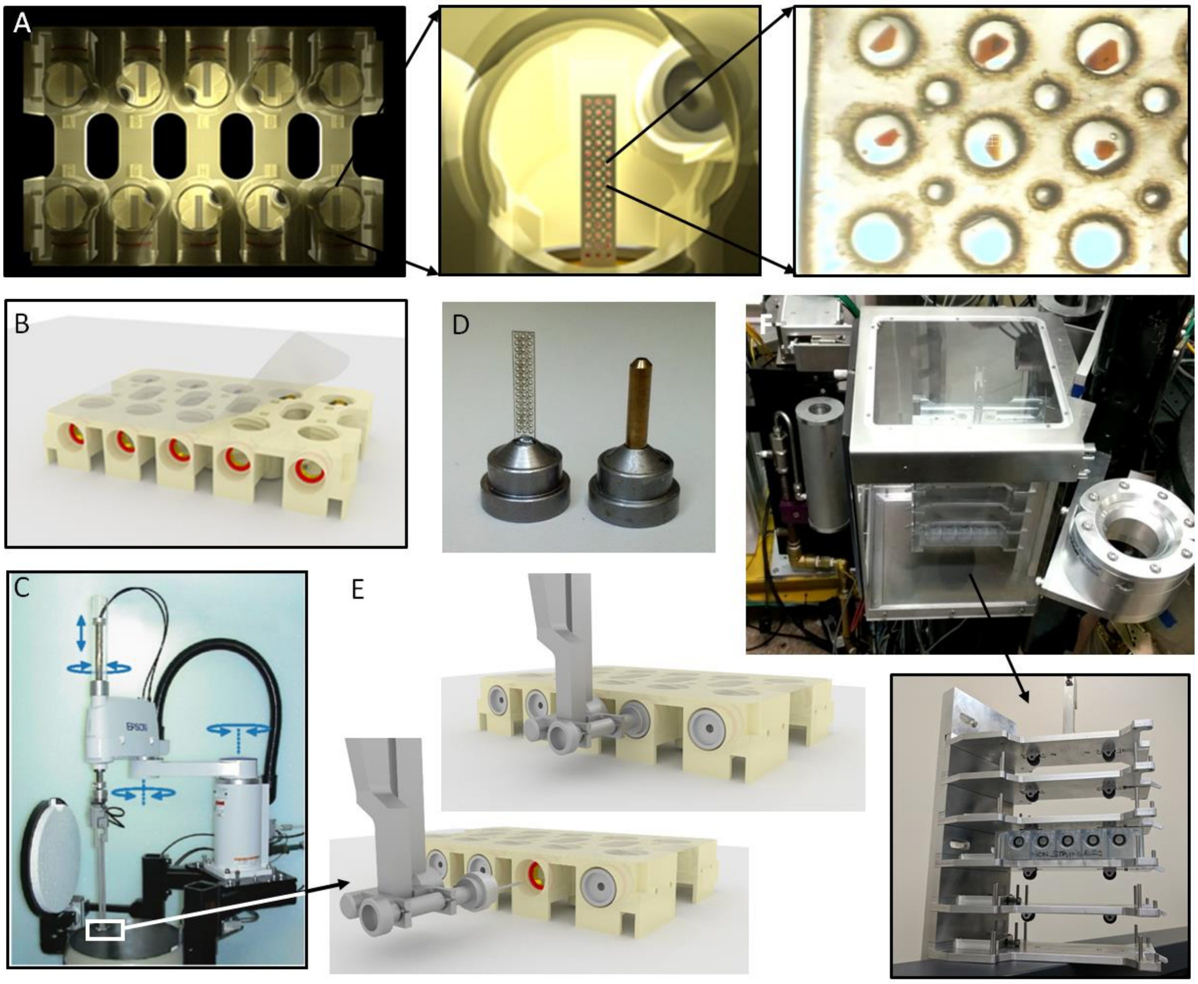

Figure S1 SMB *In-situ* crystallization plate may be used for both vapor diffusion crystallization and robotic sample mounting at SSRL and LCLS-MFX. A) The plate is shown filled with grid sample holder assemblies (ref Baxter et al). Crystals grown in grid holders within the plate may be viewed under an optical microscope. B) The plate may be sealed with tape on the top and sides for crystallization. C) Crystals may be mounted directly from the plate using the SAM robot (ref SAM). D) Grid sample holder assembly next to a copper standard pin. E) The SAM robot removes sample bases from the plate using a magnet tool. F) Five plates reside in a specialized shelf that is held inside a humidity controlled box at LCLS-MFX.

Table S1 Systems studied by multi-shot goniometer femtosecond crystallography.

MR = molecular replacement.

**Protein**	**XFEL instrument**	**XFEL beam parameters (wavelength, pulse duration, flux, beam size)**	**Data collection parameters (number of crystals and images, step size)**	**Results**	**Reference**
Photosystem II (S1 state)	SACLA BL3	10 keV, 10 fs pulses, $2.6x10^{10}$ ph/pulse, 4x2.5 μm^2 beam (20 mm behind focus)	82 crystals (1.2 x 0.5 x 0.2 mm3), 2058 images, 50 μm steps, 0.2 ° rotation	Structure by MR at 1.95 Å resolution, 96% complete, with accurate Mn-Mn distances	(Suga *et al.*, 2014)
Cytochrome c oxidase	SACLA BL3	10 keV, 10 fs, $3.5x10^{10}$ ph/pulse, 1.8x1.2 μm^2 beam	76 crystals, 1396 images, 50 μm steps, 0.1° rotation	Structure by MR at 1.9 Å resolution, with correct peroxide bond length	(Hirata *et al.*, 2014)
CpI [Fe-Fe]-hydrogenase	LCLS XPP	9 keV, 40 fs pulses, 10^{12} ph/pulse, 50 μm beam	5 crystals (1-3 mm needles), 162 images, 11° attenuated oscillation on first position for strategy. Helical data collection: 70 μm steps, 0.5° rotation	Structure by MR at 1.6 Å resolution, 92% complete ($P4_22_12$)	(Cohen *et al.*, 2014)
Syt1-SNARE complex	LCLS XPP	9 keV, 40 fs pulses, 10^{12} ph/pulse, 30 μm beam	148 crystals, 578 images (final selection 72 crystals, 309 images), 100 μm steps	Structure by MR at 3.5 Å resolution, 66% complete (limited by available beamtime)	(Zhou *et al.*, 2015)

Cyclophiline A enzyme	LCLS<br>XPP	9.5 keV, 10 µm beam	71 crystals, 1239 images (772 indexed), 25-40 µm steps, RT in paratone	Structure by MR at 1.75 Å resolution, 96% complete in high resolution shell<br>($P2_12_12_1$)	(Keedy *et al.*, 2015)
Peroxydase ferryl center	LCLS<br>XPP	9.43-9.49 keV, 3x3-15x15 µm beam, 25-40 fs pulses	Helical data collection: 50-60 µm steps, 0.5° rotation. In total 371 images (263 indexed) from 34 crystals (0.15-1 mm)	Structure by MR at 1.5 Å resolution, 97% complete ($P2_12_12_1$)	(Chreifi *et al.*, 2015)
Copper nitrite reductase *Ax*NiR	SACLA BL3 EH4	10 keV, 10 fs, $3x10^{10}$ ph/pulse, 3.0x1.9 μm^2 beam (15 mm behind focus)	64 crystals, 4403 images (3656 good hits), 50 µm steps, 0.1° rotation	Structure by MR at1.6A resolution cut-off, 100% completeness (R3 space group)	(Halsted *et al.*, 2018)

Table S2 Systems studied by fixed target SFX by the multiple small crystals approach

Protein	XFEL instrument	XFEL beam parameters (energy, flux)	Support, method, sample parameters	Number of images, hit rate	Results	Reference
REP24	LCLS CXI	8 keV, 30 fs pulses, 10^9-10^{11} ph/pulse,1.3 µm beam, 5-10 Hz	Silicon nitride solid support (long rectangular windows), 12x5 µm crystals, vacuum	610 images (38% with Bragg peaks) from 6 supports, 100 µm steps	Diffraction at 2.5 Å resolution, 1 image/s or 0.3 hit/s (including chip alignment)	(Hunter *et al.*, 2014)
beta-adrenoreceptor complex	LCLS XPP	9 keV, 40 fs pulses, 10^{12} ph/pulse, 5 and 30 µm beam, 1 Hz	microgrids (cryo), 20-100 µm crystals		Diffraction at 2.3 Å, dataset could be collected at 2.8 Å (limited beamtime), better than synchrotron resolution	(Cohen *et al.*, 2014)
PolII-TFIIB- NAS complex	LCLS XPP	9.6 keV, 30 fs pulses, 10^{12} ph/pulse, 20 µm beam, 1 Hz	raster on micromeshes and in-situ microgrid (cryo), 50 µm crystals		Diffraction to 3.3 Å (limited beamtime), better than synchrotron resolution	(Cohen *et al.*, 2014)
Myoglobin	LCLS XPP	9.5 keV, 40 fs pulses, 10^{12} ph/pulse, 50 µm beam	32 microgrids coated in Paratone N oil (cryo)	932 crystals (637 indexed)	Structure by MR at 1.36 Å, >90% complete	(Cohen *et al.*, 2014)

Bacteriorhodopsin (2D)	LCLS CXI	8.4-8.8 keV, 30 fs pulses, 2 mJ 100%, 0.3 µm beam	Silicon nitride support (array). RT, sugar embedded.	324 images acquired in 6 min (30% few lattices, 5% single lattice).	Diffraction at better than 7 Å	(Frank *et al.*, 2014; Pedrini *et al.*, 2014; Casadei *et al.*, 2018)
Bacteriorhodopsin (2D)	LCLS CXI	8.8 keV, 30 fs pulses, 2 mJ 100%, 0.3 µm beam	TEM metal mesh, plastic wafer (SU8 photoresist) covered with carbon film (up to 20 nm thickness). RT, sugar embedded.		Proof of principle (limited beamtime)	(Feld *et al.*, 2015)
anthrax toxin protective antigen construct	LCLS CXI	8.8 keV, 30 fs pulses, <1% trans-mission ,0.3 µm beam	plastic wafer and grid (SU8 photoresist) covered with polyvinyl formal thin film. RT, <10 µm crystals embedded in Paratone N oil		Proof of principle (limited beamtime)	(Feld *et al.*, 2015)
Hen egg white lysozyme	LCLS XPP	80 fs pulses, 3 µm beam, $2x10^{12}$ ph/pulse	15 µm crystals trapped in microfluidic chip, RT. 0-30° tilting to compensate preferential orientation of crystals	Stills data collection at SSRL beamline 12-2: 1 image per crystal with 0.02° rotation, 265 images (232 integrated)	Diffraction to 1.8 Å resolution at LCLS. Strong PDMS ring at 7.5 Å. stills data collection at synchrotron yields structure by	(Lyubimov *et al.*, 2015)

					MR at 2.5 Å	
Myoglobin-CO	LCLS XPP	8.1 keV, 36 fs pulses, 10 µm beam, monochro-matic beam, 120 Hz	30-60 µm crystals trapped in windows array of a Si chip, RT. Pump laser 540 nm, 2.5 mJ/mm^2	2289 wells of a single chip, each 1 'dark' (10 images accumulated at 10% X-ray beam) and 1 'light' image (unattenuated) with delays from 200 fs to 32 ps after pump laser pulse, resulting in resp. 57 ('dark') and 116 ('light') indexed images.	Diffraction at 2.0 Å Resolution. Pump-probe proof-of-principle.	(Mueller *et al.*, 2015)
Myoglobin-CO	LCLS XPP	9.58 keV, 20 µm beam, 10^{10} ph/pulse, 10 Hz	20x20x40-70 µm crystals trapped in wells of Si chip (RT), chip mapped with microspectrometer to identify crystal-containing wells	8957 indexable patterns, 85% total hit rate, 45% indexable hit rate	Structure solution at 1.9 Å.	(Oghbaey *et al.*, 2016)
Hen egg white lysozyme	LCLS XPP	9 keV, 40 fs pulses, 3 µm beam	3-layer patterned chip. Chip tilted ±44° in 2° steps for completeness.	Stills data collection at APS: 324 crystals on 8 chips	1.3 Å resolution.	(Murray *et al.*, 2015)

Lysozyme, photosystem II, mouse perforin, Pol II-TFIIB-TB-25 complex, myoglobin, Influenza A M2 protein transmembrane domain peptide	LCLS XPP	9.5 keV, 2.6 mJ, <50 fs X-ray pulses, 10 µm beam	Polycarbonate microgrids with 75 ports backed with 5 µm polycarbonate film, various crystal types and sizes tested. Step size of 50 µm for grids over 400 µm ports. Tilting ±20°. RT and cryo	See (Cohen *et al.*, 2014)	See (Cohen *et al.*, 2014)	(Baxter *et al.*, 2016)
Hen egg white lysozyme	-	-	4 µm crystals deposited on silicon membranes (10-30 µm thick, pores of 2–5 µm with a pitch of 20 µm), cryo	I24 at DLS, 139 datasets of 3°, 0.05° per image, 110 datasets indexed	2.1 Å resolution, MR	(Roedig *et al.*, 2015)
CPV18 polyhedrin	-	-	4 µm crystals deposited on silicon membranes (10-30 µm thick, pores of 2–5 µm with a pitch of 20 µm), cryo	I24 at DLS, 51 datasets of 5°, 0.05° per image, 23 datasets indexed	1.5 Å resolution, MR	(Roedig *et al.*, 2015)
Porcine insulin	-	-	20-50 µm crystals deposited on silicon membranes (10 µm thick, pores of 8 µm with a	I03 at DLS, 70° total range of rotation per single crystal	1.9 Å resolution	(Roedig *et al.*, 2016)

			pitch of 10 µm), RT			
Picornavirus bovine enterovirus 2	LCLS XPP	9.5 keV, 3 x 3 µm beam, 40% transmission, 30 Hz (shot every 4th hole)	8 µm crystals deposited on silicon membranes (10 µm thick, pores of 4-8 µm with a pitch of 10 µm), RT	5 chips, 8812 images, 352 indexed, hit rate 2-9%	Structure solution by MR at 2.3 Å (detector limited)	(Roedig *et al.*, 2017)
CPV18 polyhedrin	LCLS XPP	9.5 keV, 3 x 3 µm beam, 40% transmission, 120 Hz	microcrystals deposited on silicon membranes (10 µm thick, pores of 4-8 µm with a pitch of 10 µm), cryo	1 chip, 19’000 images, 16’700 indexing solutions (multiple lattices), 70% hit rate	Structure solution by MR at 2.4 Å (detector limited)	(Roedig *et al.*, 2017)
Env-BG18 complexes	LCLS MFX	9.5 keV X-ray pulses with 40 fs duration and a 5-µm beam	~75 µm x 75 µm x 50 µm crystal mounted on specialized loops for prelocated data collection at MFX	627 images (570 indexed)	Structure by MR at 3.8 Å resolution, 99.1% complete	(Barnes *et al.*, 2018)
Lysozyme and haemoglobin-CO	SACLA BL2	7.3 keV X-ray pulses, full transmission, 1.4x1.6 µm FWHM, 30 Hz	7 µm crystals in mother liquor sandwiched between 2 sheets of 2.5 µm Mylar foils, spacings of 50 - 250 µm	10-30% hit rate. 14 000 indexed images for lysozyme (78% ind. rate). 26 000 images for Hb.CO (38% indexing rate)	Structures by MR at 2.1 and 2.2 Å (100% complete)	(Doak *et al.*, 2018)

Barnes, C. O., Gristick, H. B., Freund, N. T., Escolano, A., Lyubimov, A. Y., Hartweger, H., West, A. P., Cohen, A. E., Nussenzweig, M. C. & Bjorkman, P. J. (2018). *Nature Communications*. **9**, 1251.

Baxter, E. L., Aguila, L., Alonso-Mori, R., Barnes, C. O., Bonagura, C. a., Brehmer, W., Brunger, A. T., Calero, G., Caradoc-Davies, T. T., Chatterjee, R., Degrado, W. F., Fraser, J. S., Ibrahim, M., Kern, J., Kobilka, B. K., Kruse, A. C., Larsson, K. M., Lemke, H. T., Lyubimov, A. Y., Manglik, A., McPhillips, S. E., Norgren, E., Pang, S. S., Soltis, S. M., Song, J., Thomaston, J., Tsai, Y., Weis, W. I., Woldeyes, R. a., Yachandra, V., Yano, J., Zouni, A. & Cohen, A. E. (2016). *Acta Crystallographica Section D Structural Biology*. **72**, 1–10.

Casadei, C. M., Tsai, C.-J., Barty, A., Hunter, M. S., Zatsepin, N. A., Padeste, C., Capitani, G., Benner, W. H., Boutet, S., Hau-Riege, S. P., Kupitz, C., Messerschmidt, M., Ogren, J. I., Pardini, T., Rothschild, K. J., Sala, L., Segelke, B., Williams, G. J., Evans, J. E., Li, X.-D., Coleman, M., Pedrini, B. & Frank, M. (2018). *IUCrJ*. **5**, 103–117.

Chreifi, G., Baxter, E. L., Doukov, T., Cohen, A. E., Mcphillips, S. E. & Song, J. (2015). 1–6.

Cohen, A. E., Soltis, S. M., González, A., Aguila, L., Alonso-Mori, R., Barnes, C. O., Baxter, E. L., Brehmer, W., Brewster, A. S., Brunger, A. T., Calero, G., Chang, J. F., Chollet, M., Ehrensberger, P., Eriksson, T. L., Feng, Y., Hattne, J., Hedman, B., Hollenbeck, M., Holton, J. M., Keable, S., Kobilka, B. K., Kovaleva, E. G., Kruse, A. C., Lemke, H. T., Lin, G., Lyubimov, A. Y., Manglik, A., Mathews, I. I., McPhillips, S. E., Nelson, S., Peters, J. W., Sauter, N. K., Smith, C. a, Song, J., Stevenson, H. P., Tsai, Y., Uervirojnangkoorn, M., Vinetsky, V., Wakatsuki, S., Weis, W. I., Zadvornyy, O. a, Zeldin, O. B., Zhu, D. & Hodgson, K. O. (2014). *Proc. Natl. Acad. Sci. U. S. A.* **111**, 17122–17127.

Doak, R. B., Nass Kovacs, G., Gorel, A., Foucar, L., Barends, T. R. M., Grünbein, M. L., Hilpert, M., Kloos, M., Roome, C. M., Shoeman, R. L., Stricker, M., Tono, K., You, D., Ueda, K., Sherrell, D. A., Owen, R. L. & Schlichting, I. (2018). *Acta Crystallographica Section D Structural Biology*. **74**, 1000–1007.

Feld, G. K., Heymann, M., Benner, W. H., Pardini, T., Tsai, C.-J., Boutet, S., Coleman, M. a., Hunter, M. S., Li, X., Messerschmidt, M., Opthalage, A., Pedrini, B., Williams, G. J., Krantz, B. a., Fraden, S., Hau-Riege, S., Evans, J. E., Segelke, B. W. & Frank, M. (2015). *Journal of Applied Crystallography*. **48**, 1072–1079.

Frank, M., Carlson, D. B., Hunter, M. S., Williams, G. J., Messerschmidt, M., Zatsepin, N. a, Barty, A., Benner, W. H., Chu, K., Graf, A. T., Hau-Riege, S. P., Kirian, R. a, Padeste, C., Pardini, T., Pedrini, B., Segelke, B., Seibert, M. M., Spence, J. C. H., Tsai, C.-J., Lane, S. M., Li, X.-D., Schertler, G., Boutet, S., Coleman, M. & Evans, J. E. (2014). *IUCrJ*. **1**, 95–100.

Halsted, T. P., Yamashita, K., Hirata, K., Ago, H., Ueno, G., Tosha, T., Eady, R. R., Antonyuk, S. V., Yamamoto, M. & Hasnain, S. S. (2018). *IUCrJ*. **5**, 22–31.

Hirata, K., Shinzawa-Itoh, K., Yano, N., Takemura, S., Kato, K., Hatanaka, M., Muramoto, K., Kawahara, T., Tsukihara, T., Yamashita, E., Tono, K., Ueno, G., Hikima, T., Murakami, H., Inubushi, Y., Yabashi, M., Ishikawa, T., Yamamoto, M., Ogura, T., Sugimoto, H., Shen, J.-R., Yoshikawa, S. & Ago, H. (2014). *Nature Methods*. **11**, 734–736.

Hunter, M. S., Segelke, B., Messerschmidt, M., Williams, G. J., Zatsepin, N. a, Barty, A., Benner, W. H., Carlson, D. B., Coleman, M., Graf, A., Hau-Riege, S. P., Pardini, T., Seibert, M. M., Evans, J., Boutet, S. & Frank, M. (2014). *Scientific Reports*. **4**, 6026.

Keedy, D. A., Kenner, L. R., Warkentin, M., Woldeyes, R. A., Hopkins, J. B., Thompson, M. C., Brewster, A. S., Van Benschoten, A. H., Baxter, E. L., Uervirojnangkoorn, M., McPhillips, S. E., Song, J., Alonso-Mori, R., Holton, J. M., Weis, W. I., Brunger, A. T., Soltis, S. M., Lemke, H., Gonzalez, A., Sauter, N. K., Cohen, A. E., van den Bedem, H., Thorne, R. E. & Fraser, J. S. (2015). *ELife*. **4**,.

Lyubimov, A. Y., Murray, T. D., Koehl, A., Araci, I. E., Uervirojnangkoorn, M., Zeldin, O. B., Cohen, A. E., Soltis, S. M., Baxter, E. L., Brewster, A. S., Sauter, N. K., Brunger, A. T. & Berger, J. M. (2015). *Acta Crystallographica Section D Biological Crystallography*. **71**, 928–940.

Mueller, C., Marx, a., Epp, S. W., Zhong, Y., Kuo, a., Balo, a. R., Soman, J., Schotte, F., Lemke, H. T., Owen, R. L., Pai, E. F., Pearson, A. R., Olson, J. S., Anfinrud, P. a., Ernst, O. P. & Dwayne Miller, R. J. (2015). *Structural Dynamics*. **2**, 054302.

Murray, T. D., Lyubimov, A. Y., Ogata, C. M., Vo, H., Uervirojnangkoorn, M., Brunger, A. T. & Berger, J. M. (2015). *Acta Crystallographica Section D Biological Crystallography*. **71**, 1987–1997.

Oghbaey, S., Sarracini, A., Ginn, H. M., Pare-Labrosse, O., Kuo, A., Marx, A., Epp, S. W., Sherrell, D. A., Eger, B. T., Zhong, Y., Loch, R., Mariani, V., Alonso-Mori, R., Nelson, S., Lemke, H. T., Owen, R. L., Pearson, A. R., Stuart, D. I., Ernst, O. P., Mueller-Werkmeister, H. M. & Miller, R. J. D. (2016). *Acta Crystallographica Section D Structural Biology*. **72**, 944–955.

Pedrini, B., Tsai, C., Capitani, G., Padeste, C., Hunter, M. S., Zatsepin, N. A., Barty, A., Benner, W. H., Boutet, S., Feld, G. K., Hau-Riege, S. P., Kirian, R. A., Kupitz, C., Messerschmitt, M., Ogren, J. I., Pardini, T., Segelke, B., Williams, G. J., Spence, J. C. H., Abela, R., Coleman, M., Evans, J. E., Schertler, G. F. X., Frank, M. & Li, X. (2014). *Philosophical Transactions of the Royal Society of London. Series B, Biological Sciences*. **369**, 20130500.

Roedig, P., Duman, R., Sanchez-Weatherby, J., Vartiainen, I., Burkhardt, A., Warmer, M., David, C., Wagner, A. & Meents, A. (2016). *Journal of Applied Crystallography*. **49**, 968–975.

Roedig, P., Ginn, H. M., Pakendorf, T., Sutton, G., Harlos, K., Walter, T. S., Meyer, J., Fischer, P., Duman, R., Vartiainen, I., Reime, B., Warmer, M., Brewster, A. S., Young, I. D., Michels-Clark, T., Sauter, N. K., Kotecha, A., Kelly, J., Rowlands, D. J., Sikorsky, M., Nelson, S., Damiani, D. S., Alonso-Mori, R., Ren, J., Fry, E. E., David, C., Stuart, D. I., Wagner, A. & Meents, A. (2017). *Nature Methods*.

Roedig, P., Vartiainen, I., Duman, R., Panneerselvam, S., Stübe, N., Lorbeer, O., Warmer, M., Sutton, G., Stuart, D. I., Weckert, E., David, C., Wagner, A. & Meents, A. (2015). *Sci. Rep.* **5**, 10451.

Suga, M., Akita, F., Hirata, K., Ueno, G., Murakami, H., Nakajima, Y., Shimizu, T., Yamashita, K., Yamamoto, M., Ago, H. & Shen, J. (2014). *Nature*. **517**, 99–103.

Zhou, Q., Lai, Y., Bacaj, T., Zhao, M., Lyubimov, A. Y., Uervirojnangkoorn, M., Zeldin, O. B., Brewster, A. S., Sauter, N. K., Cohen, A. E., Soltis, S. M., Alonso-Mori, R., Chollet, M., Lemke, H. T., Pfuetzner, R. a., Choi, U. B., Weis, W. I., Diao, J., Südhof, T. C. & Brunger, A. T. (2015). *Nature*. **525**, 62–67.
